# Characteristics of physicians working at geriatric health service facilities in Japan, 1996–2016

**DOI:** 10.1371/journal.pone.0250589

**Published:** 2021-04-27

**Authors:** Masatoshi Ishikawa, Masao Iwagami, Nanako Tamiya

**Affiliations:** 1 Takemi Program in International Health, Harvard T.H. Chang School of Public Health, Boston, Massachusetts, United States of America; 2 Department of Health Services Research, Faculty of Medicine, University of Tsukuba, Tsukuba, Ibaraki, Japan; 3 Health Services Research & Development Center, University of Tsukuba, Tsukuba, Ibaraki, Japan; 4 Department of Non-Communicable Disease Epidemiology, London School of Hygiene and Tropical Medicine, London, United Kingdom; China Medical University Hospital, TAIWAN

## Abstract

**Background:**

In Japan, which has the most rapidly aging population worldwide, the number of geriatric health service facilities (GHSFs) has been increasing. GHSF physicians play significant roles in integrated care for the elderly. However, little is known about the temporal trends of physicians working in GHSFs.

**Objectives:**

We aim to examine temporal trends in the characteristics of GHSF physicians and identify physician factors associated with starting work at GHSFs.

**Design:**

Cohort study.

**Setting and participants:**

Physicians responding to biennial national physician census surveys conducted by the Ministry of Health, Labour and Welfare of Japan from 1996 to 2016. The response rate was approximately 90%.

**Methods:**

We estimated temporal trends in the number, proportion, and characteristics of GHSF physicians. A multivariable logistic regression analysis identified physician factors associated with starting work at GHSFs 10 years after 1996–2006 and 2006–2016 among physicians not working in GHSFs at baseline (1996 and 2006).

**Results:**

GHSF physicians rapidly increased in the first decade from 1,127 (0.47%) in 1996 to 2,891 (1.04%) in 2006; this trend then slowed and was almost proportional to that of all physicians, reaching 3,345 (1.05%) in 2016. GHSF physicians aged ≥65 years increased from 61.2% in 1996 to 68.5% in 2016, while those aged <40 years decreased from 13.8% to 1.9%. The sex ratio (male vs. female physicians) increased from 5.7 in 1996 to 6.4 in 2016. Physician factors associated with starting to work at GHSFs included older age, female sex, rural area, working at hospitals, and majored in internal medicine and surgery specialties.

**Conclusions:**

The proportion of GHSF physicians among all physicians has stagnated, and GHSF physicians are aging. Facilitating the transition of younger physicians from clinical practice in hospitals to GHSFs will increase the number of GHSF physicians and improve the quality of care in GHSFs.

## Introduction

Population aging is one of the most important public health issues worldwide. Owing to decreasing birth rates and increasing life expectancy, the Japanese population has been aging rapidly in recent decades. The average life expectancy of Japanese men and women was 81 and 87.1 years, respectively, according to the most recent estimates recorded in 2016 [1]. The proportion of people aged ≥65 years was 27.3% in 2016, the highest globally [1]. Accordingly, considerations around how to care for elderly people, both inside and outside of hospitals, is becoming increasingly important in society.

In Japan, geriatric health service facilities (GHSFs) were first established in 1987 under the Health Service Law for the Aged. They provide temporal intermediate care, which includes medical and welfare services, for elderly individuals when they need rehabilitation before returning home from the hospital [2, 3]. Elderly people are admitted to GHSFs from the hospital after their acute medical conditions become stable, and they stay until they are ready to return home [4]. A GHSF is more similar to a skilled nursing facility than a hospital and mainly funded by public long-term care insurance [4]. Physicians working at a GHSF are typically salaried for more than 32 hours a week. Most GHSF physicians provide out-of-hours calls, and do not have other employment such as a family medicine office or hospital/community geriatric medicine practice [4].

According to the Health Service Law for the Aged, GHSFs are required to assign at least one full-time physician per institution, employed as the director of GHSFs, and at least one physician per 100 beds [4]. The requirements for physicians working at GHSFs do not include professional qualifications in areas such as geriatrics or family medicine [4].

The number of GHSFs has been increasing since 1987 due to rapid population aging and increasing demand for intermediate care for elderly people discharged from hospitals [3]. As of October 2017, 4,322 GHSFs with 372,679 beds have been established in Japan [5].

In a super-aging society, GHSF physicians are expected to play a key role in integrated care, a major global health issue highlighted by the World Health Organization [6]. However, the temporal trend in the number, proportion (among all Japanese physicians), and characteristics of physicians working in Japan’s GHSFs has, to date, not been investigated. Further, although it is expected that many physicians will decide to work for GHSFs during the course of their career, physician factors associated with starting their career in GHSFs are unknown. Hence, this information will be useful for creating a strategy to secure the quality and quantity of physicians employed in GHSFs.

In this study, we used national physician census surveys, conducted over two decades (1996–2016) by the Japanese Ministry of Health, Labour and Welfare, to evaluate recent changes in the number, proportion, and characteristics of physicians working in GHSFs in Japan and to identify the physician factors associated with starting work at GHSFs.

As there is insufficient information on the distribution and characteristics of GHSF physicians, the present study provided information that may contribute to investigations into the circumstances of GHSF physicians and measures for securing their services in the future.

## Methods

### Data source

To conduct this study, we used a questionnaire available from the Japanese Ministry of Health, Labour and Welfare (http://www.mhlw.go.jp/toukei/sonota/chousahyo.html). We used individual physician data spanning two decades (1996–2016) from the Survey of Physicians, Dentists, and Pharmacists, which is a national census survey conducted every two years by the Japanese Ministry of Health, Labour and Welfare. In Japan, the Medical Practitioners Act requires all physicians to report their status every two years. The response rate in this study was approximately 90% [7]. The study was approved by the Institutional Review Board of the Harvard T.H. Chan School of Public Health (No. 18–1422). We obtained the required permits and approvals that apply to foreign researchers to do this study in Japan from the Japanese Ministry of Health, Labour and Welfare (No. H30-0814-1). The data were stored and managed by Wellness Co., Ltd. located in Japan. Informed consent from individual physicians was waived because of the anonymous nature of the data.

The survey data included the physicians’ ID (registration number), age, sex, length of medical practice and qualifications, whether they qualified as a physician over the age of 30 years (i.e., indicating roughly whether they entered medical college directly after high school), type of workplace (type of municipality and medical institution), and specialty (internal medicine, surgery, pediatrics, or other). The physicians’ actual IDs were matched and replaced with random identifying numbers to ensure data anonymization. For this sample, physicians who chose “working at GHSFs” from a predetermined list in the survey questionnaire were considered full-time GHSF physicians because the questionnaire asks about where they work full time. In terms of geography, 344 secondary medical areas in Japan were divided into three groups (urban, intermediate, and rural) in 2016 based on the combination of population size and population density.

### Data analysis

First, the physicians were categorized into overall (total) and working/not working at GHSFs in 2006 and 2016; they were further categorized according to age (<40, 40–54, 55–64, or ≥65 years). We calculated the proportion of full-time GHSF physicians among all physicians who responded to the census survey during the two decades, from 1996 to 2016 (every two years). We also described the temporal changes in the characteristics of GHSF physicians, including sex ratio (i.e., male vs. female physicians), the number of years since receiving their qualification as a physician, and working location (i.e., rural, intermediate, or urban).

Second, we conducted a cohort study to identify physician factors associated with starting work at GHSFs 10 years later; the first decade spanned 1996–2006, and the second 2006–2016. We separated the first and second decades mainly because we found that the trend showing an increase in the number and proportion of GHSF physicians was different between the first and second decades. In the first cohort (1996–2006), we included all physicians who responded to the physician census survey and *were not* working in GHSFs in 1996. The outcome of interest was working at GHSFs 10 years later in 2006, while the exposures of interest were physician characteristics in 1996: age category (<40, 40–54, 55–64, or ≥65 years), sex, whether qualified as a physician over 30 years of age (yes or no), working area (urban, intermediate, or rural), type of institution (clinic, academic hospital, nonacademic hospital, or other), and specialty (internal medicine, surgery, pediatrics, or other). We did not include the length of physician qualification, which was strongly correlated with age. We described the baseline characteristics in 1996 for physicians overall and by the outcome status (i.e., physicians working in GHSFs or not in 2006) with chi-square tests; this was followed by a multivariable logistic regression analysis. We repeated the analysis for the second cohort (2006–2016).

In addition, we investigated the temporal trend in the rate of continuing to work in GHSFs (among physicians currently working in GHSFs) and their characteristics to identify factors that may motivate physicians to continue working at GHSFs. We defined “retention rate” as the number of physicians working at GHSFs in the subsequent survey (e.g., in 1998) divided by the number working at GHSFs in the current survey (e.g., in 1996) among physicians responding to both surveys (e.g., in 1996 and 1998), and we demonstrated the trend from 1996–1998 through 2014–2016. Finally, we conducted a cohort study to identify GHSF physician factors associated with continuing work in GHSFs 10 years later. We followed the same method as with the first cohort study. However, in the first cohort (1996–2006), we included physicians who *were* working in GHSFs in 1996. The outcome of interest was still working in GHSFs 10 years later, while the exposures of interest were GHSF physician characteristics in 1996: age category (<40, 40–54, 55–64, or ≥65 years), sex (male or female), whether qualified as a physician over 30 years of age (yes or no), and working area (urban, intermediate, or rural). The analysis was repeated for the second cohort (2006–2016).

P values of less than 0.05 were considered significant. STATA 15.1 (StataCorp 2017 Stata Statistical Software: Release 15. College Station, TX: StataCorp LLC) was used for all statistical analyses.

## Results

**Fig 1:**
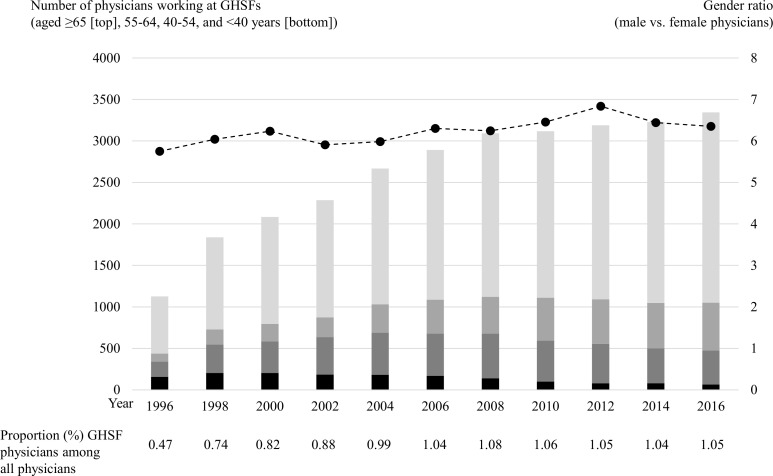
Temporal trend in the number (overall and by age category), proportion, and sex ratio of physicians working at GHSFs in Japan from 1996 to 2016. GHSFs = geriatric health service facilities.

Fig 1 shows the number (overall and by age category), proportion, and sex ratio of GHSF physicians over the two decades from 1996 to 2016 (Table 1 presents the detailed statistics including years of physician qualification and working area). Both the number and proportion of GHSF physicians rapidly increased in the first decade from 1,127 (0.47%) in 1996 to 2,891 (1.04%) in 2006, while the increase was slow and almost proportional to that of all physicians in Japan in the second decade, reaching 3,345 (1.05%) in 2016. The GHSF physicians were aging: the proportion of GHSF physicians aged ≥65 years of all GHSF physicians in the same year increased from 61.2% in 1996 to 68.5% in 2016, while that of GHSF physicians aged <40 years decreased from 13.8% in 1996 to 1.9% in 2016. Similarly, the years of physician experience tended to be longer over the two decades: the proportion of GHSF physicians with ≥45 years of experience increased from 41.0% in 1996 to 52.5% in 2016 (Table 1). The sex ratio (male vs. female physicians) increased from 5.7 in 1996 to 6.4 in 2016. The distribution of working area categories also changed over the two decades: the proportions of physicians working in urban, intermediate, and rural GHSFs were 19.8%, 65.2%, and 15.0%, respectively, in 1996; whereas in 2006, the corresponding values were 30.5%, 56.6%, and 12.9%, respectively (Table 1).

**Table 1:** Temporal trend in the number, proportion, and characteristics of physicians working at GHSFs.

	1996 Survey	1998 Survey	2000 Survey	2002 Survey	2004 Survey	2006 Survey	2008 Survey	2010 Survey	2012 Survey	2014 Survey	2016 Survey
Total subjects, N	1127	1838	2084	2286	2668	2891	3093	3117	3189	3230	3345
(% of all physicians)	0.5	0.7	0.8	0.9	1.0	1.0	1.1	1.1	1.1	1.0	1.0
Age, n (%)											
≦39	156 (13.8)	204 (11.1)	204 (9.8)	183 (8.0)	182 (6.8)	168 (5.8)	139 (4.5)	100 (3.2)	78 (2.4)	79 (2.4)	65 (1.9)
40–54	184 (16.3)	342 (18.6)	381 (18.3)	451 (19.7)	506 (19.0)	508 (17.6)	539 (17.4)	493 (15.8)	477 (15.0)	421 (13.0)	410 (12.3)
55–64	97 (8.6)	182 (9.9)	211 (10.1)	239 (10.5)	343 (12.9)	410 (14.2)	443 (14.3)	517 (16.6)	536 (16.8)	547 (16.9)	576 (17.2)
≧65	690 (61.2)	1110 (60.4)	1288 (61.8)	1413 (61.8)	1637 (61.4)	1805 (62.4)	1972 (50.8)	2007 (64.4)	2098 (65.8)	2183 (67.6)	2294 (68.6)
Gender, n (%)											
Male	960 (85.2)	1577 (85.8)	1796 (86.2)	1955 (85.5)	2286 (85.7)	2495 (86.3)	2666 (86.2)	2699 (86.6)	2782 (87.2)	2796 (86.6)	2890 (86.4)
Female	167 (14.8)	261 (14.2)	288 (13.8)	331 (14.5)	382 (14.3)	396 (13.7)	427 (13.8)	418 (13.4)	407 (12.8)	434 (13.4)	455 (13.6)
Ratio (male vs. female)	5.7	6.0	6.2	5.9	6.0	6.3	6.2	6.5	6.9	6.4	6.4
Years of experience, n (%)											
0–14	190 (16.9)	251 (13.7)	257 (12.3)	222 (9.7)	230 (8.6)	188 (6.5)	178 (5.8)	122 (3.9)	99 (3.1)	104 (3.2)	89 (2.7)
15–29	163 (14.5)	301 (16.4)	340 (16.3)	423 (18.5)	501 (18.8)	550 (19.0)	581 (18.8)	567 (18.2)	539 (16.9)	492 (15.2)	473 (14.1)
30–44	312 (27.7)	521 (28.3)	587 (28.2)	630 (27.6)	730 (27.4)	807 (27.9)	878 (28.4)	950 (30.5)	932 (29.2)	942 (29.2)	1026 (30.7)
≧45	462 (41.0)	765 (41.6)	900 (43.2)	1011 (44.2)	1207 (45.2)	1346 (46.6)	1456 (47.1)	1478 (47.4)	1619 (50.8)	1692 (52.4)	1757 (52.5)
Workplace, n (%)											
Urban	223 (19.8)	417 (22.7)	548 (26.3)	629 (27.5)	732 (27.4)	808 (27.9)	972 (31.4)	931 (29.9)	1023 (32.1)	1062 (32.9)	1020 (30.5)
Intermediate	735 (65.2)	1132 (61.6)	1219 (58.5)	1308 (57.2)	1531 (57.3)	1689 (58.4)	1689 (54.6)	1754 (56.3)	1744 (54.7)	1752 (54.2)	1894 (56.6)
Rural	169 (15.0)	289 (15.7)	317 (15.2)	349 (15.3)	405 (15.2)	394 (13.6)	432 (14.0)	432 (13.9)	422 (13.2)	416 (12.9)	431 (12.9)

GHSF = geriatric health service facility

Table 2 presents the baseline characteristics of all physicians (except for those already working in GHSFs) included in the 1996–2006 and 2006–2016 cohorts, overall and separately by the outcome status (i.e., physicians working/not working in GHSFs, 10 years later). In the 1996–2006 cohort (N = 194242), 2304 (1.19%) physicians who had started working at GHSFs in 1996 were still working at GHSFs in 2006, while in the 2006–2016 cohort (N = 227514), 2149 (0.94%) physicians who had started working at GHSFs in 2006 were working at GHSFs in 2016. In both cohorts, physicians working in GHSFs 10 years later were older and more likely to be male, qualified as a physician over 30 years of age, more likely to have majored in internal medicine and surgery specialties, and more likely to work in rural areas and nonacademic hospitals than physicians not working in GHSFs. In the multivariable logistic regression analyses (Table 3), the physician factors associated with starting to work at GHSFs 10 years later included older age, being female, qualified as a physician over 30 years of age (only in the 2006–2016 cohort), worked in rural areas, worked at hospitals (vs. clinics), and majored in internal medicine and surgery specialties (vs. other specialties). Regarding the fitting of the logistic regression model, the P value (Prob > ChiSq) was less than 0.01; hence, the estimated model was evaluated as appropriate.

**Table 2:** Baseline characteristics of physicians (except for physicians already working at GHSFs) in the 1996–2006 and 2006–2016 cohorts overall and by outcome status.

Baseline characteristics*	The 1996–2006 cohort				The 2006–2016 cohort			
	Overall	Physicians working at GHSFs in 2006	Physicians not working at GHSFs in 2006	P value	Overall	Physicians working at GHSFs in 2016	Physicians not working at GHSFs in 2016	P value
	(N = 194242)							
					(N = 227514)			
						(N = 2149)		
							(N = 225365)	
			(N = 191938)					
		(N = 2304)						
	n (%)	n (%)	n (%)		n (%)	n (%)	n (%)	
Age category (years)				<0.001				<0.001
<40	86768 (45)	330 (14)	86438 (45)		84417 (37)	176 (8)	83971 (37)	
40–54	66596 (34)	478 (21)	66118 (34)		93601 (41)	465 (22)	93136 (41)	
55–64	20960 (11)	759 (33)	20251 (11)		32046 (14)	774 (36)	31272 (14)	
≥65	19918 (10)	737 (32)	19181 (10)		17720 (8)	734 (34)	16986 (8)	
Sex				<0.001				<0.001
Male	169788 (87)	2053 (89)	167735 (87)		189290 (83)	1914 (89)	187376 (83)	
Female	24454 (13)	251 (11)	24203 (13)		38224 (17)	235 (11)	37989 (17)	
Qualified as a physician over 30 years of age				<0.001				<0.001
No	143359 (74)	1602 (70)	141757 (74)		171147 (75)	1539 (72)	169608 (75)	
Yes	50883 (26)	702 (30)	50181 (26)		56367 (25)	610 (28)	55757 (25)	
Working area				<0.001				<0.001
Urban	87367 (45)	793 (34)	86574 (45)		103952 (46)	717 (33)	103235 (46)	
Intermediate	91863 (47)	1253 (54)	90610 (47)		107461 (47)	1190 (55)	106271 (47)	
Rural	15012 (8)	258 (11)	14754 (8)		16101 (7)	242 (11)	15859 (7)	
Type of institution				<0.001				<0.001
Clinic	59396 (31)	741 (32)	58655 (31)		71263 (31)	690 (32)	70573 (31)	
Academic hospital	36509 (19)	170 (7)	36339 (19)		40470 (18)	101 (5)	40369 (18)	
Nonacademic hospital	91838 (47)	1223 (53)	90615 (47)		107727 (47)	1183 (55)	106544 (47)	
Other	6499 (3)	170 (7)	6329 (3)		8054 (4)	175 (8)	7879 (3)	
Specialty				<0.001				<0.001
Internal medicine	74553 (39)	1044 (45)	73509 (38)		81109 (36)	1001 (47)	80108 (36)	
Surgery	30833 (16)	415 (18)	30418 (16)		30527 (13)	364 (17)	30163 (13)	
Pediatrics	11269 (6)	55 (2)	11214 (6)		12219 (5)	64 (3)	12155 (5)	
Other	77587 (41)	790 (34)	76797 (40)		103659 (46)	720 (34)	102939 (46)	

CI = confidence interval, OR = odds ratio, GHSF = geriatric health service facility

*Characteristics of physicians in 1996 of the 1996–2006 cohort and those in 2006 of the 2006–2016 cohort.

**Table 3:** Multivariable logistic regression analysis to identify physician factors associated with starting to work in GHSFs 10 years later.

Baseline characteristics*	The 1996–2006 cohort		The 2006–2016 cohort	
	Adjusted OR (95% CI)	P value	Adjusted OR (95% CI)	P value
Age category (years)				
<40	1 (reference)	-	1 (reference)	-
40–54	2.26 (1.96–2.61)	<0.01	2.49 (2.08–2.97)	<0.01
55–64	14.53 (12.66–16.68)	<0.01	13.83 (11.64–16.43)	<0.01
≥65	17.59 (15.1–20.34)	<0.01	26.83 (22.47–32.04)	<0.01
Sex				
Male	1 (reference)	-	1 (reference)	-
Female	1.27 (1.11–1.46)	<0.01	1.19 (1.03–1.37)	0.02
Qualified as a physician over 30 years of age				
No	1 (reference)	-	1 (reference)	-
Yes	1.02 (0.93–1.12)	0.63	1.12 (1.02–1.24)	0.02
Working area				
Urban	1 (reference)	-	1 (reference)	-
Intermediate	1.49 (1.36–1.63)	<0.01	1.42 (1.29–1.56)	<0.01
Rural	1.72 (1.48–1.98)	<0.01	1.68 (1.45–1.95)	<0.01
Type of institution				
Clinic	1 (reference)	-	1 (reference)	-
Academic hospital	2.01 (1.68–2.42)	<0.01	1.36 (1.09–1.70)	<0.01
Nonacademic hospital	3.06 (2.76–3.38)	<0.01	2.66 (2.40–2.94)	<0.01
Other	4.92 (4.09–5.93)	<0.01	5.07 (4.21–6.11)	<0.01
Specialty				
Internal medicine	1.51 (1.36–1.67)	<0.01	1.75 (1.57–1.94)	<0.01
Surgery	1.47 (1.29–1.67)	<0.01	1.72 (1.50–1.97)	<0.01
Pediatrics	0.57 (0.43–0.75)	<0.01	0.81 (0.63–1.06)	0.12
Other	1 (reference)	-	1 (reference)	-

CI = confidence interval, OR = odds ratio, GHSF = geriatric health service facility

*Characteristics of physicians in 1996 of the 1996–2006 cohort and those in 2006 of the 2006–2016 cohort.

**Fig 2:**
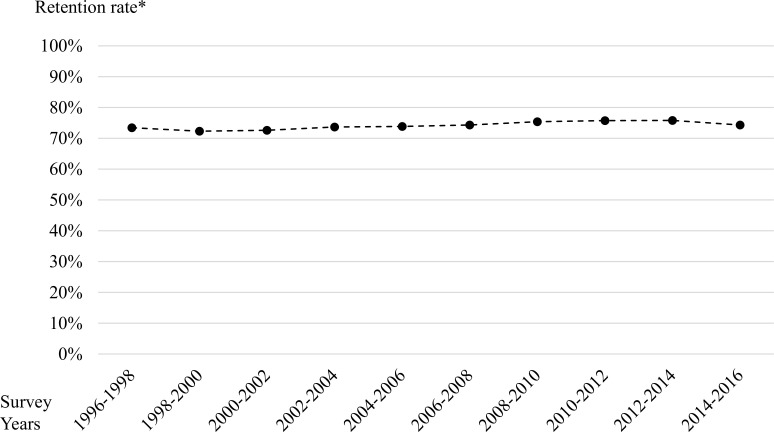
Temporal trend in the retention rate of physicians working at GHSFs in Japan from 1996 to 2016. *Retention rate was defined as the number of physicians working at GHSFs in the subsequent survey (e.g., in 1998) divided by the number of physicians working at GHSFs in the current survey (e.g., in 1996) among physicians responding to both surveys (e.g., in 1996 and 1998).

Fig 2 shows the temporal change in the retention rate from 1996 to 2016. The retention rate was almost constant (nearly 75%) over both decades. Table 4 shows the baseline characteristics of physicians working in GHSFs included in the 1996–2006 and 2006–2016 cohorts, overall and separately by the outcome status. In the 1996–2006 cohort (N = 616), of the physicians working at GHSFs in 1996, 244 (60%) continued to work at GHSFs in 2006, while 134 (22%), 194 (31%), and 44 (7%) were working in clinics, hospitals, and other institutions, respectively. In the 2006–2016 cohort (N = 1,431), of the physicians working at GHSFs in 2006, 789 (55%) continued to work at GHSFs in 2016, while 249 (17%), 334 (23%), and 59 (4%) were working in clinics, hospitals, and other institutions, respectively. In both cohorts, physicians still working in GHSFs 10 years later were older than physicians not working in GHSFs. In multivariable logistic regression analyses (Table 5), the physician factors associated with continuing to work at GHSFs included older age and female sex.

**Table 4:** Baseline characteristics of physicians already working at GHSFs from 1996–2006 and 2006–2016 cohorts overall and by outcome status.

Baseline characteristics*	The 1996–2006 cohort				The 2006–2016 cohort			
	Overall	Physicians working at GHSFs in 2006	Physicians not working at GHSFs in 2006	P value	Overall	Physicians working at GHSFs in 2016	Physicians not working at GHSFs in 2016	P value
	(N = 616)							
					(N = 1431)			
						(N = 789)		
							(N = 642)	
			(N = 372)					
		(N = 244)						
	n (%)	n (%)	n (%)		n (%)	n (%)	n (%)	
Age category (years)				<0.001				<0.001
<40	135 (22)	25 (10)	110 (30)		141 (10)	47 (6)	94 (15)	
40–54	154 (25)	61 (25)	93 (25)		443 (31)	209 (26)	234 (36)	
55–64	62 (10)	27 (11)	35 (9)		285 (20)	163 (21)	122 (19)	
≥65	265 (43)	131 (54)	134 (36)		562 (39)	370 (47)	192 (30)	
Sex				<0.001				<0.001
Male	504 (82)	196 (80)	308 (83)		1166 (81)	645 (82)	521 (81)	
Female	112 (18)	48 (20)	64 (17)		268 (19)	144 (18)	121 (19)	
Qualified as a physician over 30 years of age				<0.001				<0.001
No	420 (68)	160 (66)	260 (70)		1016 (71)	569 (72)	447 (70)	
Yes	196 (32)	84 (34)	112 (30)		415 (29)	220 (28)	195 (30)	
Working area				<0.001				<0.001
Urban	126 (20)	49 (20)	77 (21)		388 (27)	204 (26)	184 (29)	
Intermediate	409 (66)	174 (71)	235 (63)		857 (60)	480 (61)	377 (59)	
Rural	81 (13)	21 (9)	60 (16)		186 (13)	105 (13)	81 (13)	

**Table 5:** Multivariable logistic regression analysis to identify physician factors associated with continuing to work in GHSFs 10 years later.

Baseline characteristics*	The 1996–2006 cohort		The 2006–2016 cohort	
	Adjusted OR (95% CI)	P value	Adjusted OR (95% CI)	P value
Age category (years)				
<40	1 (reference)	-	1 (reference)	-
40–54	3.03 (1.74–5.29)	<0.01	1.94 (1.29–2.91)	<0.01
55–64	3.83 (1.92–7.61)	<0.01	3.19 (2.05–4.96)	<0.01
≥65	5.04 (2.97–8.53)	<0.01	4.66 (3.06–7.09)	<0.01
Sex				
Male	1 (reference)	-	1 (reference)	-
Female	1.79 (1.13–2.84)	0.01	1.55 (1.15–2.10)	<0.01
Qualified as a physician over 30 years of age				
No	1 (reference)	-	1 (reference)	-
Yes	1.15 (0.80–1.65)	0.44	0.96 (0.75–1.21)	0.70
Working area				
Urban	1 (reference)	-	1 (reference)	-
Intermediate	1.34 (0.88–2.05)	0.17	1.11 (0.87–1.42)	0.40
Rural	0.65 (0.35–1.24)	0.19	1.13 (0.79–1.62)	0.51

CI = confidence interval, OR = odds ratio, GHSF = geriatric health service facility

*Characteristics of physicians working in GHSFs in 1996 of the 1996–2006 cohort and those in 2006 of the 2006–2016 cohort.

## Discussion

Although the number of physicians working in GHSFs has been increasing in Japan, the proportion of GHSF physicians among all physicians has stagnated over the past decade. More importantly, GHSF physicians are aging, while the number of younger GHSF physicians has severely decreased. The physician factors associated with starting to work at GHSFs 10 years later included older age, being female, worked in rural areas, working at hospitals (vs. clinics), and majored in internal medicine and surgery specialties (vs. other specialties).

A previous study (systematic review) on medical students indicate that, according to some studies, many women are interested in geriatric medicine, but differences by age have not been observed [8]. In Japan, the reason most GHSF physicians are over 65 years old may be that they retired from the hospital and decided to work for GHSFs as a second career. Most hospitals in Japan have the same retirement system as other industries in Japan [9]; thus, medical practitioners are typically expected to retire voluntarily at age 60 or 65. Mandatory retirement practices may be responsible for the inverse relationship between years of experience and retention among certain specialists in Japan.

Furthermore, it is possible that older physicians and female physicians are more likely to enter GHSFs, and continue to work at these facilities because the work burden is low compared to hospitals, resulting in better work-life balance. According to a previous study with regard to geriatric medicine, medical students and residents cited a perceived lighter call schedule and flexibility in work hours as attractive features of this specialty [10]. In addition, physicians who originally majored in internal medicine and surgery may transition to GHSFs more easily than those from other specialties.

Securing physicians engaged in geriatric medicine is difficult in many countries [10, 11]. Researchers often note that geriatric medicine is considered to be of low prestige when physicians make decisions about their careers. A study of American medical students found that geriatric medicine did not engage their interest, and some were discouraged by what they considered the “futility” of such care [12]. Thus, there may be a need to develop educational content that enhances the characteristics of geriatric medicine.

Moreover, a study of Japanese medical students and younger physicians found that it was difficult to incorporate geriatric medicine into Japanese medical school curricula since, originally, no section had been devoted to geriatric medicine [13]. A geriatric specialist system exists as a subspecialty of internal medicine specialists, but as of 2018, the total number of such specialists is 1460, which is only 4% of the number of internal medicine specialists [14]. Thus, physicians have limited practical training for GHSF in their undergraduate or postgraduate clinical courses.

Geriatrics is becoming increasingly important not only in Japan, which is facing a super-aging society, but also worldwide [15]. To secure not only older/female but also younger physicians to start and continue working at GHSFs, there is a need for flexibility regarding when physicians can enter geriatric medicine training. In addition, strategies to promote the growth of geriatrics must include better reimbursement for physicians with geriatrics training and certification. To develop geriatrics, medical education and training of specialists and researchers should be promoted urgently as a national policy; thereby, more physicians would be recruited into GHSFs and the quality of care would improve.

Further research is needed to explain the reasons behind career choices, and further action and incentives are required to welcome and retain physicians initiating work at GHSFs. Improving and facilitating smooth transitions for younger physicians from clinical practice in hospitals to geriatric health services may be warranted to secure the required number of physicians and quality of care in GHSFs.

The strength of the present study is the large sample size and high response rate of data from the national census. However, this study has several limitations. First, the characteristics of physicians were self-reported and, therefore, misclassification may have occurred. Second, we did not obtain data to investigate GHSF physicians who were employed part-time. According to another survey, as of 2015, the number of physicians working at GHSFs full-time and part-time was 3,458 (84.5%) and 635 (15.5%), respectively [5]. In that survey, only the number of GHSF physicians was disclosed; other detailed features were not described. Third, owing to the use of secondary data, potential explanatory variables that might be associated with working at GHSFs could not be considered; these include the place of origin of the physician, graduating university, salary, and family structure [16–18]. Sociodemographic factors, lifestyles, and other potential confounding factors can contribute to GHSF physicians’ career path. Fourth, this study focused only on association and was unable to determine causality. Using interviews and questionnaires could facilitate research with a broader scope. Fifth, we divided the SMAs into three groups according to population density, but changes in classification may cause variation in the results. Sixth, the observation period was 20 years. The effects of various environmental changes such as the global economic crisis, policy changes for physician maldistribution, and population ageing were not considered.

## Conclusions and implications

Using data from the biennial national physician census surveys in Japan from 1996 to 2016, we found that the increase in the number of GHSF physicians was rapid during the first decade since 1996 and slowed considerably in the second decade. Further, the average age of GHSF physicians increased, while the number of younger GHSF physicians severely decreased. We found several physician factors such as being older and being female as factors associated with starting and continuing to work at GHSFs. Geriatrics is becoming increasingly important not only in Japan, which is facing a super-aging society, but also worldwide. To secure not only older/female but younger physicians to start and continue working at GHSFs, flexibility regarding when physicians can enter geriatric medicine training is needed.

Although there is insufficient information on the distribution and characteristics of GHSF physicians, the present study provided information that may contribute to investigations into the circumstances of GHSF physicians and measures for securing their service in the future.

## Supporting information

S1 Fig (TIFF)

S1 Table Temporal trend in the number, proportion, and characteristics of physicians working at GHSFs. (DOCX)

S2 Table Baseline characteristics of physicians already working at GHSFs in the 1996–2006 and 2006–2016 cohorts overall and by outcome status. (DOCX)

S3 Table Multivariable logistic regression analysis to identify physician factors associated with continuing to work in GHSFs 10 years later. (DOCX)
