# Supplementary figures and images for: Characteristics of physicians working at geriatric health service facilities in Japan, 1996–2016
Source: PLoS One. 2021 Apr 27;16(4):e0250589. doi: 10.1371/journal.pone.0250589 (PMC8078794; doi:10.1371/journal.pone.0250589)

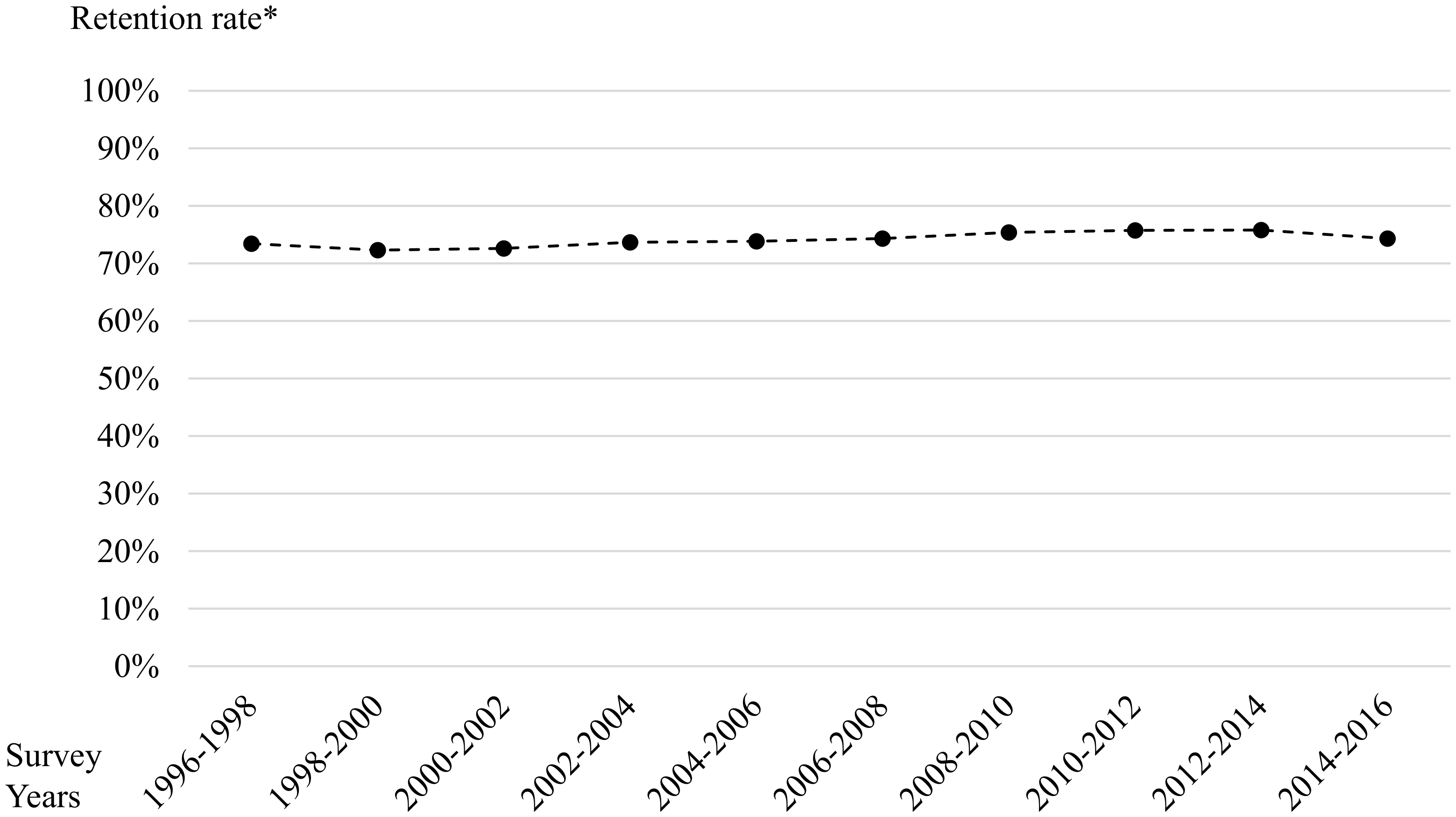

Supplement: S1 Fig — (TIFF) [file pone.0250589.s001.tiff]
