# Supplementary material for: Characteristics of physicians working at geriatric health service facilities in Japan, 1996–2016
Source: PLoS One. 2021 Apr 27;16(4):e0250589. doi: 10.1371/journal.pone.0250589 (PMC8078794; doi:10.1371/journal.pone.0250589)
Supplement: S1 Table — (DOCX) [file pone.0250589.s002.docx]

**S1 Table. Temporal trend in the number, proportion, and characteristics of physicians working at GHSFs**

|  | 1996 Survey | 1998 Survey | 2000 Survey | 2002 Survey | 2004 Survey | 2006 Survey | 2008 Survey | 2010 Survey | 2012 Survey | 2014 Survey | 2016 Survey |
| --- | --- | --- | --- | --- | --- | --- | --- | --- | --- | --- | --- |
| Total subjects, N | 1127 | 1838 | 2084 | 2286 | 2668 | 2891 | 3093 | 3117 | 3189 | 3230 | 3345 |
| (% of all physicians) | 0.5 | 0.7 | 0.8 | 0.9 | 1.0 | 1.0 | 1.1 | 1.1 | 1.1 | 1.0 | 1.0 |
| Age, n (%) |  |  |  |  |  |  |  |  |  |  |  |
| ≦39 | 156 (13.8) | 204 (11.1) | 204 (9.8) | 183 (8.0) | 182 (6.8) | 168 (5.8) | 139 (4.5) | 100 (3.2) | 78 (2.4) | 79 (2.4) | 65 (1.9) |
| 40–54 | 184 (16.3) | 342 (18.6) | 381 (18.3) | 451 (19.7) | 506 (19.0) | 508 (17.6) | 539 (17.4) | 493 (15.8) | 477 (15.0) | 421 (13.0) | 410 (12.3) |
| 55–64 | 97 (8.6) | 182 (9.9) | 211 (10.1) | 239 (10.5) | 343 (12.9) | 410 (14.2) | 443 (14.3) | 517 (16.6) | 536 (16.8) | 547 (16.9) | 576 (17.2) |
| ≧65 | 690 (61.2) | 1110 (60.4) | 1288 (61.8) | 1413 (61.8) | 1637 (61.4) | 1805 (62.4) | 1972 (50.8) | 2007 (64.4) | 2098 (65.8) | 2183 (67.6) | 2294 (68.6) |
| Gender, n (%) |  |  |  |  |  |  |  |  |  |  |  |
| Male | 960 (85.2) | 1577 (85.8) | 1796 (86.2) | 1955 (85.5) | 2286 (85.7) | 2495 (86.3) | 2666 (86.2) | 2699 (86.6) | 2782 (87.2) | 2796 (86.6) | 2890 (86.4) |
| Female | 167 (14.8) | 261 (14.2) | 288 (13.8) | 331 (14.5) | 382 (14.3) | 396 (13.7) | 427 (13.8) | 418 (13.4) | 407 (12.8) | 434 (13.4) | 455 (13.6) |
| Ratio (male vs. female) | 5.7 | 6.0 | 6.2 | 5.9 | 6.0 | 6.3 | 6.2 | 6.5 | 6.9 | 6.4 | 6.4 |
| Years of experience, n (%) |  |  |  |  |  |  |  |  |  |  |  |
| 0–14 | 190 (16.9) | 251 (13.7) | 257 (12.3) | 222 (9.7) | 230 (8.6) | 188 (6.5) | 178 (5.8) | 122 (3.9) | 99 (3.1) | 104 (3.2) | 89 (2.7) |
| 15–29 | 163 (14.5) | 301 (16.4) | 340 (16.3) | 423 (18.5) | 501 (18.8) | 550 (19.0) | 581 (18.8) | 567 (18.2) | 539 (16.9) | 492 (15.2) | 473 (14.1) |
| 30–44 | 312 (27.7) | 521 (28.3) | 587 (28.2) | 630 (27.6) | 730 (27.4) | 807 (27.9) | 878 (28.4) | 950 (30.5) | 932 (29.2) | 942 (29.2) | 1026 (30.7) |
| ≧45 | 462 (41.0) | 765 (41.6) | 900 (43.2) | 1011 (44.2) | 1207 (45.2) | 1346 (46.6) | 1456 (47.1) | 1478 (47.4) | 1619 (50.8) | 1692 (52.4) | 1757 (52.5) |
| Workplace, n (%) |  |  |  |  |  |  |  |  |  |  |  |
| Urban | 223 (19.8) | 417 (22.7) | 548 (26.3) | 629 (27.5) | 732 (27.4) | 808 (27.9) | 972 (31.4) | 931 (29.9) | 1023 (32.1) | 1062 (32.9) | 1020 (30.5) |
| Intermediate | 735 (65.2) | 1132 (61.6) | 1219 (58.5) | 1308 (57.2) | 1531 (57.3) | 1689 (58.4) | 1689 (54.6) | 1754 (56.3) | 1744 (54.7) | 1752 (54.2) | 1894 (56.6) |
| Rural | 169 (15.0) | 289 (15.7) | 317 (15.2) | 349 (15.3) | 405 (15.2) | 394 (13.6) | 432 (14.0) | 432 (13.9) | 422 (13.2) | 416 (12.9) | 431 (12.9) |

GHSF = geriatric health service facility
