# Supplementary material for: Characteristics of physicians working at geriatric health service facilities in Japan, 1996–2016
Source: PLoS One. 2021 Apr 27;16(4):e0250589. doi: 10.1371/journal.pone.0250589 (PMC8078794; doi:10.1371/journal.pone.0250589)
Supplement: S2 Table — (DOCX) [file pone.0250589.s003.docx]

**S2 Table. Baseline characteristics of physicians already working at GHSFs in the 1996**–**2006 and 2006**–**2016 cohorts overall and by outcome status**

| Baseline characteristics* | The 1996–2006 cohort | | | | The 2006–2016 cohort | | | |
| --- | --- | --- | --- | --- | --- | --- | --- | --- |
|  | Overall  (N = 616) | Physicians working at GHSFs in 2006  (N = 244) | Physicians  not working at GHSFs in 2006  (N = 372) | P value | Overall  (N = 1431) | Physicians working at GHSFs in 2016  (N = 789) | Physicians  not working at GHSFs in 2016  (N = 642) | P value |
|  | n (%) | n (%) | n (%) |  | n (%) | n (%) | n (%) |  |
| Age category (years) |  |  |  | <0.001 |  |  |  | <0.001 |
| <40 | 135 (22) | 25 (10) | 110 (30) |  | 141 (10) | 47 (6) | 94 (15) |  |
| 40–54 | 154 (25) | 61 (25) | 93 (25) |  | 443 (31) | 209 (26) | 234 (36) |  |
| 55–64 | 62 (10) | 27 (11) | 35 (9) |  | 285 (20) | 163 (21) | 122 (19) |  |
| ≥65 | 265 (43) | 131 (54) | 134 (36) |  | 562 (39) | 370 (47) | 192 (30) |  |
| Sex |  |  |  | <0.001 |  |  |  | <0.001 |
| Male | 504 (82) | 196 (80) | 308 (83) |  | 1166 (81) | 645 (82) | 521 (81) |  |
| Female | 112 (18) | 48 (20) | 64 (17) |  | 268 (19) | 144 (18) | 121 (19) |  |
| Qualified as a physician over 30 years of age |  |  |  | <0.001 |  |  |  | <0.001 |
| No | 420 (68) | 160 (66) | 260 (70) |  | 1016 (71) | 569 (72) | 447 (70) |  |
| Yes | 196 (32) | 84 (34) | 112 (30) |  | 415 (29) | 220 (28) | 195 (30) |  |
| Working area |  |  |  | <0.001 |  |  |  | <0.001 |
| Urban | 126 (20) | 49 (20) | 77 (21) |  | 388 (27) | 204 (26) | 184 (29) |  |
| Intermediate | 409 (66) | 174 (71) | 235 (63) |  | 857 (60) | 480 (61) | 377 (59) |  |
| Rural | 81 (13) | 21 (9) | 60 (16) |  | 186 (13) | 105 (13) | 81 (13) |  |

CI = confidence interval, OR = odds ratio, GHSF = geriatric health service facility

*Characteristics of physicians in 1996 of the 1996–2006 cohort and those in 2006 of the 2006–2016 cohort.
