# Supplementary material for: Characteristics of physicians working at geriatric health service facilities in Japan, 1996–2016
Source: PLoS One. 2021 Apr 27;16(4):e0250589. doi: 10.1371/journal.pone.0250589 (PMC8078794; doi:10.1371/journal.pone.0250589)
Supplement: S3 Table — (DOCX) [file pone.0250589.s004.docx]

**S3 Table. Multivariable logistic regression analysis to identify physician factors associated with continuing to work in GHSFs 10 years later**

| Baseline characteristics* | The 1996–2006 cohort | | The 2006–2016 cohort | |
| --- | --- | --- | --- | --- |
|  | Adjusted OR (95% CI) | P value | Adjusted OR (95% CI) | P value |
| Age category (years) |  |  |  |  |
| <40 | 1 (reference) | - | 1 (reference) | - |
| 40–54 | 3.03 (1.74–5.29) | <0.01 | 1.94 (1.29–2.91) | <0.01 |
| 55–64 | 3.83 (1.92–7.61) | <0.01 | 3.19 (2.05–4.96) | <0.01 |
| ≥65 | 5.04 (2.97–8.53) | <0.01 | 4.66 (3.06–7.09) | <0.01 |
| Sex |  |  |  |  |
| Male | 1 (reference) | - | 1 (reference) | - |
| Female | 1.79 (1.13–2.84) | 0.01 | 1.55 (1.15–2.10) | <0.01 |
| Qualified as a physician over 30 years of age |  |  |  |  |
| No | 1 (reference) | - | 1 (reference) | - |
| Yes | 1.15 (0.80–1.65) | 0.44 | 0.96 (0.75–1.21) | 0.70 |
| Working area |  |  |  |  |
| Urban | 1 (reference) | - | 1 (reference) | - |
| Intermediate | 1.34 (0.88–2.05) | 0.17 | 1.11 (0.87–1.42) | 0.40 |
| Rural | 0.65 (0.35–1.24) | 0.19 | 1.13 (0.79–1.62) | 0.51 |

CI = confidence interval, OR = odds ratio, GHSF = geriatric health service facility

*Characteristics of physicians working in GHSFs in 1996 of the 1996–2006 cohort and those in 2006 of the 2006–2016 cohort.
